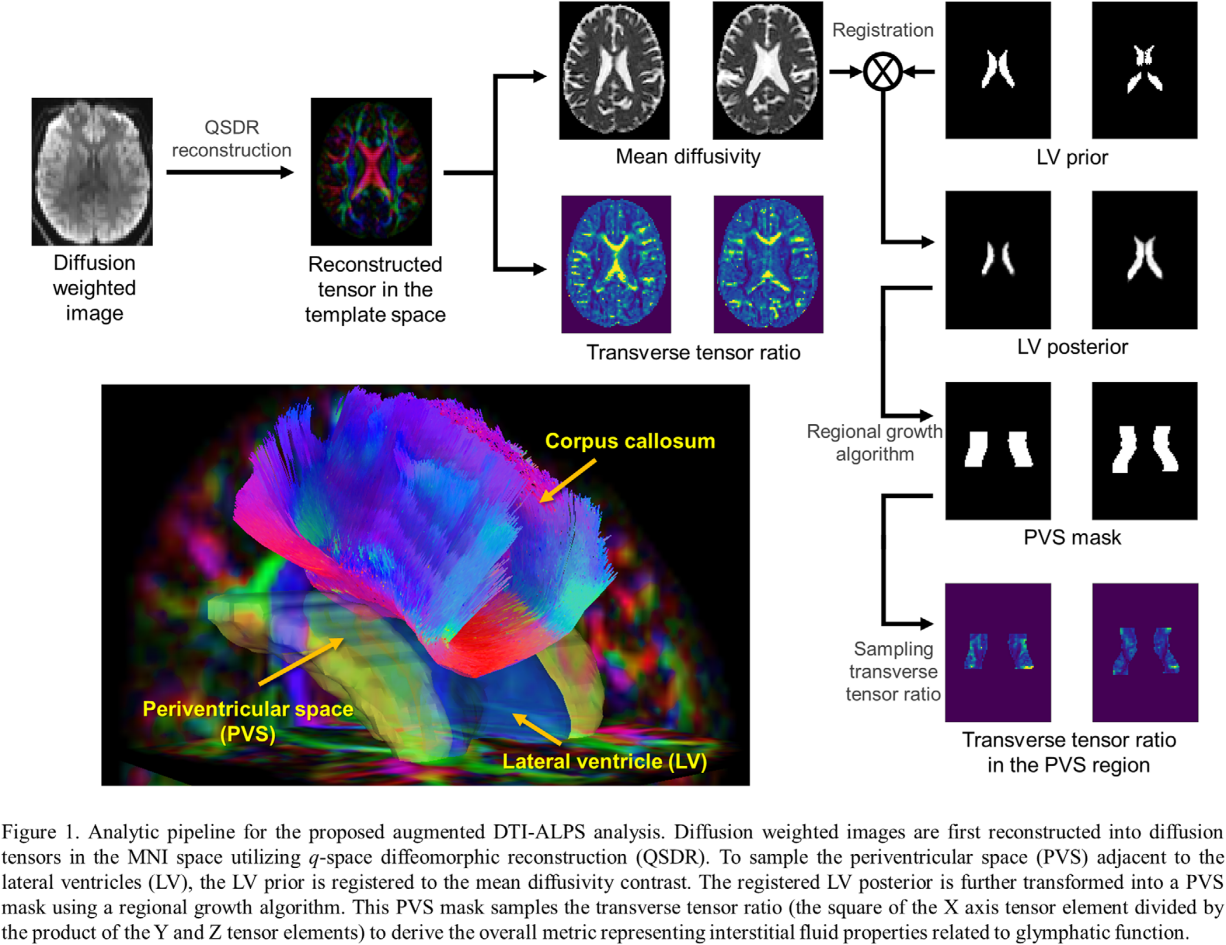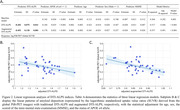# Markers of Interstitial Fluid Dynamics from Diffusion MRI Reveal Association with Amyloid Deposition in Cognitively Normal Older Adults

**DOI:** 10.1002/alz.093872

**Published:** 2025-01-09

**Authors:** Chang‐Le Chen, Hecheng Jin, Noah Schweitzer, Davneet S Minhas, Victor L Villemagne, Shaolin Yang, Howard J Aizenstein, Minjie Wu

**Affiliations:** ^1^ Department of Bioengineering, University of Pittsburgh, Pittsburgh, PA USA; ^2^ University of Pittsburgh, Pittsburgh, PA USA

## Abstract

**Background:**

Glymphatic clearance dysfunction within the perivascular space has been implicated in Alzheimer’s Disease (AD) pathology. Diffusion MRI‐based techniques such as DTI‐ALPS can quantify perivascular interstitial fluid (ISF) dynamics, yet their links with other early AD markers in the cognitively normal elderly population remain underexplored. Here, we investigated associations between DTI‐ALPS and cerebral ß‐amyloid (Aß) burden measured with Pittsburgh Compound B (PiB)‐PET. We also evaluated an augmented version of DTI‐ALPS to enhance sensitivity and better reflect the ISF diffusivity potentially associated with glymphatic function.

**Methods:**

Diffusion tensor images (DTI) and PiB‐PET images were collected from 40 cognitively normal older adults (age: 74.8 [5.8] years, sex: 18 males, mini‐mental state examination (MMSE): 28.8 [1.4], APOE‐e4%: 23%). Based on the DTI (acquired on a 3T MRI scanner with 12 diffusion directions, b‐value = 1000, and voxel size in 2 mm cubic), we estimated DTI‐ALPS index that evaluates the perivascular ISF movement along the medullary conduits and also developed an algorithm to generalize the definition of the traditional DTI‐ALPS by automatically localizing the periventricular space as the target area and calculating transverse tensor ratios as the primary measure (Figure 1). PiB‐PET analysis followed our previous validated approach, and the regional standardized uptake value ratios (SUVR) representing PiB retention relative to the cerebellar gray matter were averaged to determine global cerebral Aß burden. Linear regression analysis was applied to investigate the relationship between DTI‐ALPS and global PiB‐PET SUVR.

**Results:**

The baseline model with the predictors of age, sex, MMSE and APOE4 status significantly explained the logarithmic global SUVR of PiB‐PET (Figure 2). When incorporating DTI‐ALPS indices, the baseline models were significantly improved (Likelihood‐ratio test: both p < 0.01); the explained variance increased by 15.3% and 19.3% by adding traditional and proposed augmented DTI‐ALPS indices, respectively. The models indicated that the DTI‐ALPS indices were significantly negatively associated with the global Aß burden after adjusting the covariates (Figure 2).

**Conclusion:**

We observed that higher ISF diffusivity (likely better glymphatic clearance) corresponds to lower PiB‐PET retention (lower Aß burden) in the cognitively normal older adults, suggesting the potential utility of ISF measures as a putative AD biomarker.